# Network Analysis Reveals the Combination of Controlled-Release and Regular Urea Enhances Microbial Interactions and Improves Maize Yields

**DOI:** 10.3389/fmicb.2022.825787

**Published:** 2022-06-21

**Authors:** Peng-tao Ji, Xiong Du, Jin-chao Zhou, Yujuan Peng, Xiang-ling Li, Pei-jun Tao, Yue-chen Zhang

**Affiliations:** ^1^State Key Laboratory of North China Crop Improvement and Regulation, Hebei Agricultural University, Baoding, China; ^2^State Key Laboratory of Soil Erosion and Dryland Farming on the Loess Plateau, Institute of Soil and Water Conservation, Northwest A&F University, Xianyang, China; ^3^College of Agronomy and Biotechnology, Hebei Normal University of Science and Technology, Qinhuangdao, China

**Keywords:** rhizosphere soil, controlled release urea, maize yield, microbial diversity, microbial network complexity, North China Plain

## Abstract

Increased complexity of microbial networks can contribute to increased biodiversity and multifunctionality and thus crop productivity. However, it is not clear which combination ratio of regular and controlled-release urea will increase the soil microbial community complexity and improve maize yield in the North China Plain. To address this knowledge gap, a 2-year field experiment was conducted to explore the effects of the combination of regular (U) and controlled release (S) urea ratios [no fertilizer control (CT), regular urea alone (U), controlled-release urea alone (S), controlled-release urea mixed with regular urea 3:7 (SU3), controlled-release urea mixed with regular urea 5:5 (SU5), and controlled-release urea mixed with regular urea 7:3 (SU7)] on XianYu 688 yield and its rhizosphere and bulk soil microbial community composition and network complexity at different fertility stages. The combination of controlled-release and regular urea increased the N agronomic efficiency, N partial factors productivity, maize yield, and grain number per spike, with the maximum maize yield (9,186 kg ha^–1^) being achieved when the ratio of controlled-release urea to regular urea was 3:7 (SU3, *p* < 0.05). Maize yield increased by 13% in the SU3 treatment compared to the CT treatment. Rhizosphere soil microbial diversity remained stable at the silking stage of maize while increased at the physiological maturity stage of maize, with the increasing controlled-release to regular N fertilizer ratios (from 3:7 to 7:3, *p* < 0.05). This result suggests that a combination of regular and controlled-release N fertilizer can still substantially increase soil microbial diversity in the later stages of maize growth. The combination of controlled-release and regular urea is more effective in improving microbial network total links and average degree, and N agronomic efficiency (*R*^2^ = 0.79, *p* < 0.01), N partial factor productivity (*R*^2^ = 0.79, *p* < 0.01), spikes per unit area (*R*^2^ = 0.54, *p* < 0.05), and maize yield (*R*^2^ = 0.42, *p* < 0.05) increased with the microbial network complexity. This result indicates that the higher microbial network complexity is strongly associated with the higher N agronomic efficiency and N partial factors productivity and maize yield. In conclusion, the ratio of controlled-release to regular urea at SU3 not only increases the yield of maize and N agronomic efficiency but also enhances microbial diversity and network complexity in the North China Plain.

## Introduction

As important decomposers of ecosystems, soil microorganisms are responsible for more than 90% of the decomposition of organic matter in ecosystems (Swift et al., 1979). They are involved in essential ecosystem functions (such as soil organic carbon storage, nutrient and hydrological cycling, decomposition, and terrestrial primary production) that are closely linked to global food supply and ecosystem services (Fan et al., 2020a; Jiao et al., 2021; Wang et al., 2021). For instance, soil microorganisms can mineralize mineral nutrients by releasing extracellular enzymes (Mooshammer et al., 2014), and after the microorganism dies, its cytosol enters the soil solution where it can be absorbed and used by plants to meet their vital activities (Yadav et al., 2021). Alternatively, plants can mineralize soil nutrients through the production of organic acids by their roots or induce the proliferation of microorganisms through root exudates (Jilling et al., 2021), thereby facilitating the mineralization of nutrients. In addition to their function of providing and mineralizing nutrients, soil microorganisms can also protect plants through a number of mechanisms (Yadav et al., 2021). For example, microorganisms produce glycosides that chelate iron in the soil and limit the uptake of this trace element by pathogens, hydrogen cyanide, antibiotics, hydrolytic enzymes, and the 1-aminocyclopropane-1-carboxylic acid deaminase exhibited by some specific microorganisms (Devi et al., 2020; Yadav et al., 2021). Therefore, soil microbial-plant as a system of interconnections, interactions and feedback, which in agricultural systems can improving sustainability and productivity (Mariotte et al., 2018; Sun Y. et al., 2021; Thakur et al., 2021).

In the agroecosystems, soil microbial communities coexist in multitrophic microbial food webs, which directly or indirectly affect crop health and yield (Nihorimbere et al., 2011; Fan et al., 2020b; Yu et al., 2021). Especially in the rhizosphere (root-soil interface), the zone of soil surrounding plant roots represents a hotspot of microbial and biochemical activity (York et al., 2016; Kuzyakov and Razavi, 2019; Li R. et al., 2021). Soil microorganisms are structured and form complex food webs, reflecting strong interrelationships within ecological networks, which is fundamental for characterizing species interactions and dynamics of ecosystems (Fan et al., 2021; Yuan et al., 2021). The high microbial diversity and network complexity can contribute to increased crop yields due to the important role of rhizosphere and bulk soil microorganisms in the mineralization of soil nutrients and the provision of nutrients for crop growth (Fan et al., 2020b; Li B. B. et al., 2021). However, soil microbial communities are very sensitive to soil disturbance and changes in the external environment (Wilkinson et al., 2002; Schneider et al., 2012), and changes in their composition and structure affect soil microbial enzyme secretion, respiratory metabolism, and other functions, which in turn affect soil nutrient cycling processes (Ren et al., 2021; Tekaya et al., 2021).

Numerous experiments have confirmed that fertilization management could alter the chemical properties of the soil solution, activate the microbial community, and significantly impact the microbial community structure and its complexity (Geisseler and Scow, 2014; Liu et al., 2019; Xiong et al., 2019; Fan et al., 2020a; Ji et al., 2021; Li B. B. et al., 2021; Zhang et al., 2021). For example, compared with individual applications, the combined inorganic fertilizers and cow manure led to the most resistant microbial community, which was associated with the highest levels of nutrient availability and plant production (Fan et al., 2020a). Ji et al. (2021) found that the high organic fertilizer treatments reduced the number of links in the microbial network at the genus level. Moreover, the network of organic fertilizer treatment soils contained more functionally interrelated microbial modules than soils with chemical fertilizer treatment, and the topological roles of characteristic microorganisms and key microbial organisms were significantly different between these two treatments (Gu et al., 2019). Li B. B. et al. (2021) found that a blend of regular and controlled-release urea increased the relative abundance of *Penicillium* and *Aspergillus* fungal genera and reduced the operational taxonomic unit (OTU) of nitrifying bacteria of the genus *Nitrospira* compared to regular urea treatment. The bacterial and fungi α diversity in a blend of regular and controlled-release urea treatment was lower than the regular urea treatment (Li R. et al., 2021). Lupwayi et al. (2010) found that the controlled-release urea increased microbial biomass carbon (MBC) or functional diversity more than urea in 3 site-years, but the opposite was observed in 1 site-year. This result shows that the effect of controlled-release urea and normal urea on microbial communities is influenced by the age of application of the fertilizer. Thereby, the effect of fertilizer application on microbial communities and their complexity is influenced by the type of fertilizer and its rate. Understanding the response of microbial diversity and community structural complexity to shifts in controlled-release and regular urea rates is urgent to our understanding of microbially mediated nutrient cycling processes in agroecosystems. However, there is a lack of understanding of how controlled-release urea combined with regular urea affects rhizosphere and bulk soil microbial diversity, community composition, and network complexity. The study of the mechanisms by which the diversity, stability, and complexity of soil microbial communities affect crop yield can provide a scientific basis for sustainable agricultural development.

Nitrogen (N) fertilizer (e.g., urea) has often been generously applied to increase crop biomass yield, which plays an important role in crop growth and enhances crop yield (Guo et al., 2021; Li R. et al., 2021). Although urea plays an important role in increasing crop yields, its rapid rate of decomposition means that full basal applications of N can easily cause N loss and increase the risk of environmental pollution, resulting in early crop failure and yield loss (Geisseler and Scow, 2014). However, there are difficulties in applying N fertilizer in the middle and late stages of crop life, particularly in some crops such as maize. Controlled-release urea is a new type of fertilizer that provides N to plants slowly, controls and regulates the release of N, and meets the nutritional requirements of crops (Grant et al., 2012). Compared with regular urea, controlled-release urea can reduce the damage to the crop root system in the pre-fertility stage, extend the nutrient absorption and utilization validity period, and has the advantages of N setback, improving the efficiency of N fertilizer utilization, reducing the number of manual fertilizer follow-ups, and improving economic efficiency (Blackshaw et al., 2011; Guo et al., 2017). Consequently, a blend of regular and controlled-release urea can address both the rapid loss of N fertilizer and the N requirements for later maize growth and development (Zheng et al., 2016; Zhang et al., 2021). For instance, a growing number of studies suggested that combining urea and controlled-release urea fertilizers improves maize yield and N use efficiency and reduces ammonia volatilization (Zheng et al., 2016; Zhang et al., 2019; Guo et al., 2021). However, the effect of controlled-release urea and conventional urea ratios on crop yield may vary from region to region as the effect of controlled-release urea and conventional urea ratios on crop yield is influenced by soil nutrient levels, the age of application, and microbial communities (Lupwayi et al., 2010; Li B. B. et al., 2021; Li R. et al., 2021). Currently, it is not clear what ratio of regular to controlled-release urea will promote increased maize yields and maintain healthy soil function by promoting the diversity of microbial community structure in the North China Plain. Additionally, the relationship between microbial diversity and community structural complexity and crop yield traits under changing ratios of controlled and regular urea is currently very poorly understood.

To address the above question, i.e., to characterize the effect of the ratio of regular and controlled-release urea [the treatments include no fertilizer control (CT), regular urea alone (U), controlled-release urea alone (S), controlled-release urea mixed with regular urea 3:7 (SU3), controlled-release urea mixed with regular urea 5:5 (SU5), and controlled-release urea mixed with regular urea 7:3 (SU7)] on maize yield and bacterial community structure and its network complexity, a 2-year field experiment was conducted in Xinji, Hebei, China. This study sought to reveal the mechanism of the effect of common N fertilizer and slow-acting urea application on the changes in microbial community composition and crop productivity. Therefore, the following three questions were addressed: (i) How do regular and controlled-release urea applications alone and their pairing ratios affect maize yield, spikes per unit area, grain number per spike, and hundred grains weight? (ii) How do bacterial diversity, community composition, and network complexity respond to changes in regular and controlled-release urea applications alone and their pairing ratios? (iii) What is the relationship between microbial network complexity and maize yield under N fertilizer treatments, and what ratio of regular to controlled-release urea would be more effective in improving maize yield and microbial network complexity?

## Materials and Methods

### Site Description and Experimental Design

The field experiment was performed in 2019 at the Experimental Station of Hebei Agricultural University, Xinji City, Hebei Province, China (43°31′N, 124°48′E). This area has a temperate semi-humid continental climate where the mean annual precipitation and mean annual temperature are 516.4 mm and 13.8°C. The soil texture at the research site is clay loam (ISSS Classification, International Soil Science Society). The basic physicochemical properties of soils are a bulk density of 1.47 g cm^–3^, soil organic matter of 18.47 g kg^–1^, total N of 1.25 g kg^–1^, alkali hydrolyzable N of 91.55 g kg^–1^, extractable Olsen phosphorus of 27.50 mg kg^–1^, and ammonium acetate extractable potassium of 145.79 mg kg^–1^, and pH of 7.8.

To study the effects of regular urea, controlled-release urea, and different ratios of regular urea to controlled-release urea on maize yield and soil microbial community structure at different maize growth stages, the maize variety XianYu 688 were selected as the test variety for a 2-year field experiment in 2019. The experimental design was a randomized group design with three field replications, in which the treatment plots were 8 m long and 3.6 m wide, and the maize seedlings were sown at a density of 675,000 plants hm^–2^, with 60 cm equal spacing. The fertilizer treatments included CT, U alone, S alone (the controlled-release urea was produced by Henan Xinlianxin Fertilizer Co., Ltd., with N, P_2_O_5_, and K_2_O contents of 43, 5, and 5%, respectively), SU3, SU5, and SU7, all at a rate of 180 kg hm^–2^. All were applied as a single base application before the sowing of summer maize. The same amount of phosphorus and potash was applied to each treatment, 90 kg ha^–1^ of P_2_O_5_ and 90 kg ha^–1^ of K_2_O. Other cultivation practices, such as removal of weeds, and chemical control of diseases and pests were performed using the recommended conventional approaches.

To investigate the effect of different N fertilizer types and their ratios on the microbial community structure of rhizosphere soil and bulk soil of maize at the silking and physiological maturity stage, the soil at different maize growth stages was collected. In each N fertilizer treatment plot, an “S”-shaped random sampling strategy with 5–6 small sampling locations was chosen. The soil samples from the 5–6 sampling points in each plot were mixed to obtain a representative sample of approximately 0.5 kg. A total of 72 representative samples [6 treatments × 2 stages × 3 replicates × 2 soil (rhizosphere soil and bulk soil) = 72 samples] were collected. After careful and thorough removal of plant tissues and residues from the soil, the collected samples were put into a foam box with an ice pack and moved to a refrigerator at −80°C within 24 h for DNA extraction.

### Analysis

Soil microbial DNA was extracted from 0.5 g of well-mixed soil for each sample by grinding, freeze-thawing, and sodium dodecyl sulfate (SDS)-based cell lysis and was purified using the PowerMax Soil DNA Isolation Kit (MO BIO Laboratories, Carlsbad, CA, United States). The quality of extracted DNA was evaluated based on 260/280 nm (>1.8) and 260/230 nm (>1.7) ratios, obtained using a NanoDrop ND-1000 spectrophotometer (NanoDrop Technologies Inc., Wilmington, DE, United States). The final DNA concentration was quantified using a Quant-IT Pico Green dsDNA Kit (Invitrogen Molecular Probes Inc., Oregon, United States). Although this extraction method is more labor-intensive and time-consuming, it is capable of recovering high-molecular-weight DNA with high yield and high quality from diverse representative soil samples (Yuan et al., 2021).

Primers 515F (5′-GTGCCAGCMGCCGCGGTAA-3′ and 806R (5′-GGACTACHVGGGTWTCTAAT-3′) were used to amplify the V4 region of the bacterial 16S rRNA gene. PCR amplification and tag-encoded high-throughput sequencing of the 16S gene was performed by the Novogene Company (Beijing, China) using the Illumina HiSeq platform (PE 2500). QIIME 2.0 was used to generate raw sequence data from sequencing, and all raw reads were aligned to samples according to different barcodes (Caporaso et al., 2010). Both forward and reverse primers were trimmed. Paired-end reads of sufficient length were combined with at least 30 bp overlap into full-length sequences, and the average fragment length was 253 bp using the FLASH program (Reyon et al., 2012; Zhou et al., 2020). Unqualified sequences were filtered using the Btrim program, with a window size >20 as the threshold quality score. UPARSE was used to remove chimeric sequences and classify sequences into OTUs at a similarity of 97% (Edgar et al., 2011). All singleton OTUs were removed. Alpha diversity metrics including observed OTUs, Shannon, Simpson, Chao 1, and ACE indexes were then estimated in QIIME2.0. Bacterial representative sequences were assigned taxonomic information using the SILVA database as a reference (Pruesse et al., 2007).

### Statistical Analysis

One-way ANOVA followed by the Duncan test (α = 0.05) was carried out using IBM SPSS Statistics 21 (SPSS, Chicago, IL, United States) software to test the significant differences in maize yield, spikes per unit area, grain number per spike and hundred grains weight, N agronomic efficiency, N partial factors productivity, and microbial α diversity (i.e., Shannon, Simpson, Chao1, and ACE index) at maize growth stage (silking and physiological maturity stages) under N fertilizer treatments [including CT, U, Controlled-release urea (S, and SU3, SU5, and SU7)]. Regression analysis was performed using IBM SPSS Statistics 20 software to assess the relationships among microbial network complexity maize yield, spikes per unit area, N agronomic efficiency, and N partial factors productivity under different N fertilizer treatments.

The N agronomic efficiency (Equation 1) and N partial factor productivity (Equation 2) were calculated according to methods described by Adiele et al. (2020) and Li et al. (2022):

N agronomic efficiency (kg^–1^) = (maize yield at Nx − maize yield at N0)/N rate (Equation 1)

N partial factors productivity (kg^–1^) = maize yield at Nx_/_N rate (Equation 2)

where the Nx is N treatment, N0 is an unfertilized plot, and N rate is the amount of fertilizer applied.

Network analysis is used to assess microbial community complexity (Zhou et al., 2020; Jiao et al., 2021). For the network analysis, relative abundance at the microbial phylum level was used to generate a correlation coefficient matrix. Then, a symmetric correlation matrix was constructed using interactions calculated between each of the two microbes using the Spearman correlation coefficient based on the R statistical environment. This correlation matrix was then transformed into a similar matrix by taking the absolute values. Networks were visualized using R statistical environment (Version 3.4.2, R Development Core Team, 2016) and Gephi 0.8.2.

## Results

### Changes in Maize Yield Traits Under Different N Treatment Ratios

**FIGURE 1:**
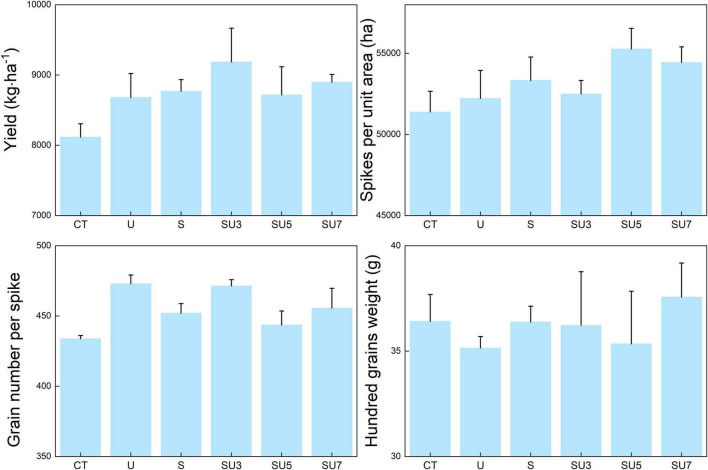
Effect of no fertilizer (CT), regular urea (U), controlled-release urea (S), and different ratios of controlled-release urea to regular urea (SU3, 3:7 ratio of controlled-release urea to regular urea; SU5, 5:5 ratio of controlled-release urea to regular urea; and SU7, 7:3 ratio of controlled-release urea to regular urea) on maize yield, spikes per unit area, grain number per spike and hundred grains weight, N agronomic efficiency, and N partial factors. Regular urea, controlled-release urea, and different ratios of regular urea and controlled-release urea are all applied at a rate of 180 kg ha^– 1^. Values are presented as the means ± standard error (SE). Lowercase and uppercase letters indicate significant differences between the nitrogen fertilizer treatment (*p* < 0.05).

Urea treatments significantly increased maize yield compared to the control treatment (Figure 1, *p* < 0.05), and the results suggested that N fertilizer application increased the crop yield in the North China Plain. However, the effect of urea application on maize yield is influenced by the ratio of controlled-release urea to regular urea. The SU3 treatment stimulates the increased maize yield compared with other N fertilizers (Figure 1, *p* < 0.05). In particular, compared to CT treatment, SU3 treatment increased maize yield by 13% (Figure 1, *p* < 0.05). This result indicates that a combination of controlled-release and regular urea is more effective in improving maize yields than the traditional regular and controlled-release urea applied separately. The results showed that the regular and controlled-release urea application increased the grain number per spike compared to the control treatment (Figure 1, *p* < 0.05). Additionally, the N agronomic efficiency and N partial factor productivity in SU3 treatment were nearly 2 times and 1.2 times higher than the CT treatment, respectively (Figure 1, *p* < 0.05). This result indicates that SU3 treatment is more effective in improving N agronomic efficiency and N partial factor productivity than CT and other treatments.

### Changes in Microbial Community Diversity and Composition Under Different N Treatments

**FIGURE 2:**
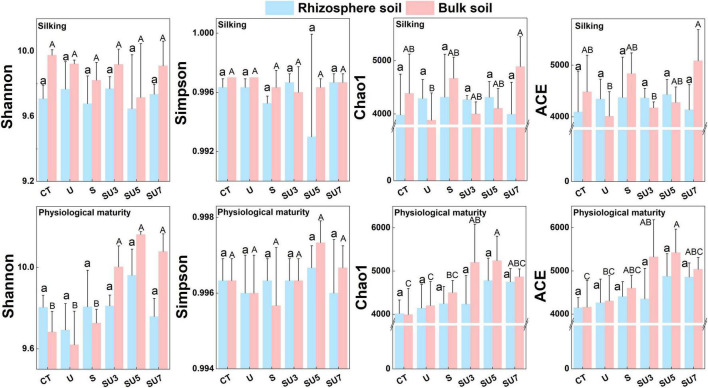
Effect of CT, U, S, SU3, SU5, and SU7 in rhizosphere and bulk soil bacterial α-diversity (Shannon, Simpson, Chao1, and ACE indexes) at different maize growing stages (i.e., silking and physiological maturity). Regular urea, controlled-release urea, and different ratios of regular urea and controlled-release urea are all applied at a rate of 180 kg ha^– 1^. Values are presented as the means ± SE. Lowercase and uppercase letters indicate significant differences between the nitrogen fertilizer treatment (*p* < 0.05).

Rhizosphere soil microbial diversity (i.e., Shannon, Simpson, Chao1, and ACE indexes) remains stable at the silking stage of maize with control and different controlled-release and regular urea ratios (Figure 2). However, results showed that the microbial diversity increased at the maize physiological maturity stage with the increasing controlled-release and regular urea ratios (controlled-release mixed with regular urea ratio increased from 3:7 to 7:3) (Figure 2, *p* < 0.05). This result demonstrated that a combination of controlled-release and regular urea can substantially increase soil microbial diversity in the later stages of maize growth (i.e., physiological maturity stage).

**FIGURE 3:**
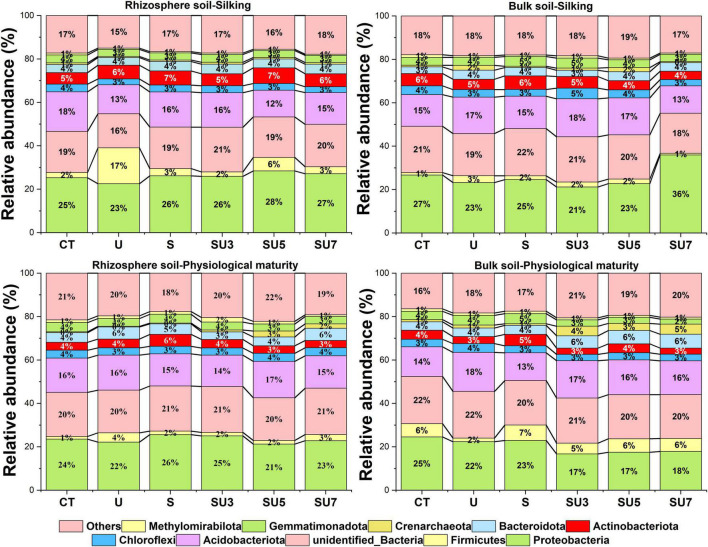
Effect of nitrogen fertilizer treatments (including CT, U, S, SU3, SU5, and SU7) on the microbial community composition of rhizosphere and bulk soil at different maize growth stages (i.e., silking and physiological maturity).

**FIGURE 4:**
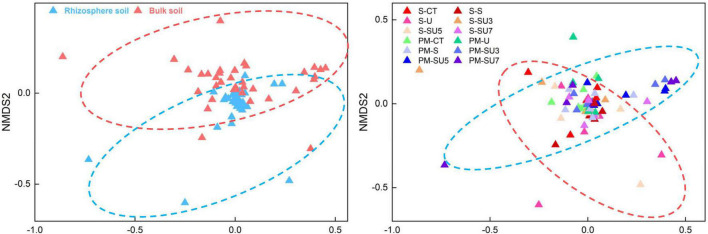
Effect of nitrogen fertilizer treatments (including CT, U, S, SU3, SU5, and SU7) on the NMDS of rhizosphere and bulk soil at different maize growth stages (i.e., silking and physiological maturity).

In all treatments, proteobacteria were the most dominant microbial taxa (Figure 3), which in the maize silking stage was slightly higher than in the physiological maturity stage. In addition, the results showed that the relative abundance of proteobacteria in bulk soil decreased at the physiological maturity stage with the increasing controlled-release and regular urea (controlled-release mixed with regular urea ratio increased from 3:7 to 7:3). The relative abundance of Firmicutes and Bacteroidota increased in bulk soil at the physiological maturity stage compared to the silking stage (Figure 3). Simultaneous, soil microbial community structure was changed with the shift rhizosphere and bulk soil, maize silking, and physiological maturity stage (Figure 4).

### Shifts in Microbial Network Complexity in Different Maize Growth Stages Under N Treatment

**FIGURE 5:**
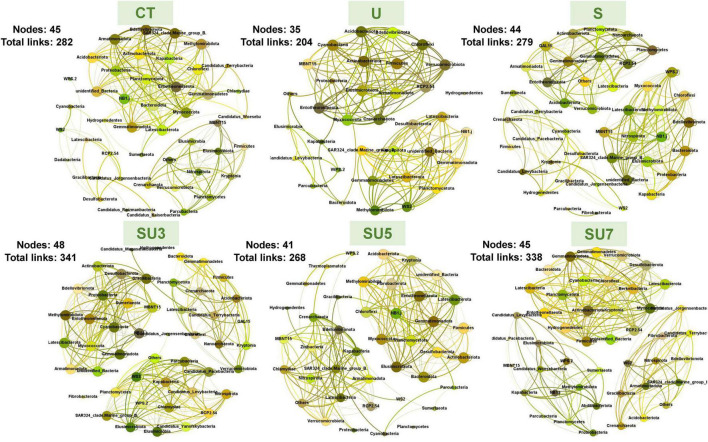
Overview of bacterial networks in the rhizosphere soil at the maize silking stage under different N fertilizer treatments (including CT, U, S, SU3, SU5, and SU7), with node size proportional to node connectivity. Node color represents various phylogenetic phyla.

**FIGURE 6:**
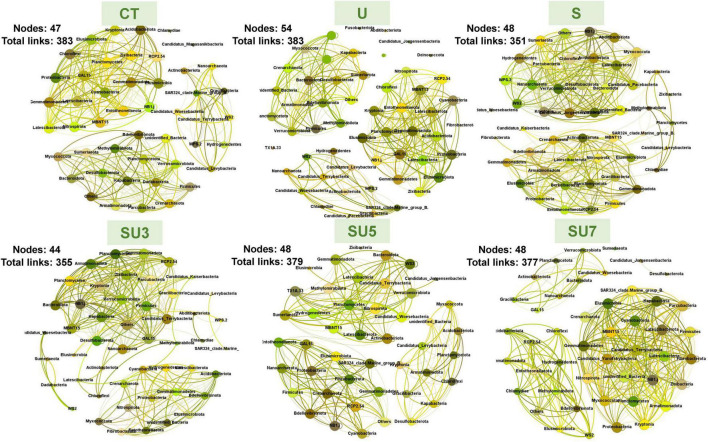
Overview of bacterial networks in the bulk soil at the maize silking stage under different N fertilizer treatments (including CT, U, S, SU3, SU5, and SU7), with node size proportional to node connectivity. Node color represents various phylogenetic phyla.

**FIGURE 7:**
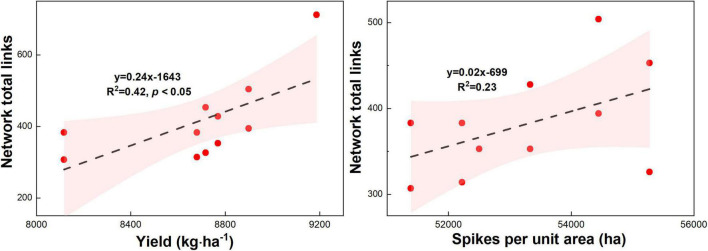
Overview of bacterial networks in the rhizosphere soil at the maize physiological maturity stage under different N fertilizer treatments (including CT, U, S, SU3, SU5, and SU7), with node size proportional to node connectivity. Node color represents various phylogenetic phyla.

**FIGURE 8:**
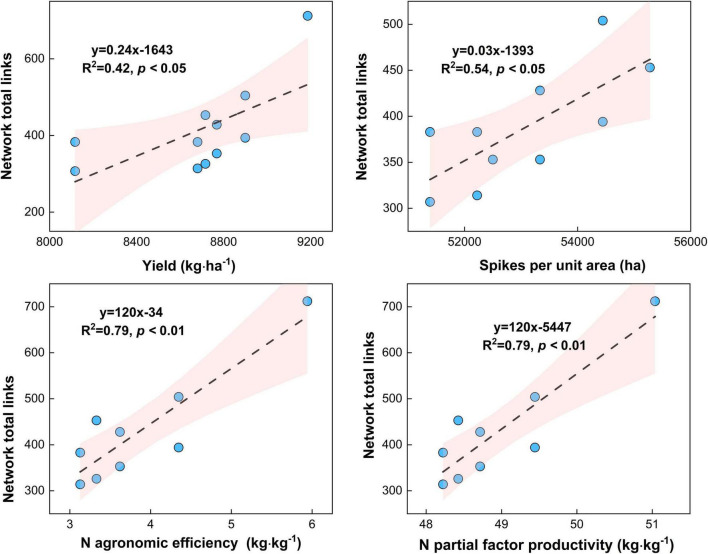
Overview of bacterial networks in the rhizosphere soil at the maize physiological maturity stage under different N fertilizer treatments (including CT, U, S, SU3, SU5, and SU7), with node size proportional to node connectivity. Node color represents various phylogenetic phyla.

The networks for rhizosphere and bulk soil at maize silking and physiological maturity stages were individually constructed and their topological properties were examined (Figures 5–8). Compared to the rhizosphere soil, the bulk soil in the maize silking stage has a higher microbial network total links and nodes (Figures 5, 6 and Table 1). In rhizosphere soil at the silking stage, the microbial network total links and nodes increased with the controlled-release to regular urea ratio, except the SU5 treatment (Figure 5). In bulk soil at the maize physiological maturity stage, the microbial network total links and average degree increased with the slow-release to regular N fertilizer ratios (Table 2). In particular, the combination of controlled-release and regular urea (i.e., SU3, 712, SU5, 453, and SU7, 504) had higher microbial network total links than the controlled-release and regular urea applied separately as well as the control treatment (Figure 8). This result suggests that the combination of controlled-release and regular urea is more effective in improving microbial network total links and an average degree in bulk soil at the maize physiological maturity stage than the traditional controlled-release and regular urea applied separately as well as the control treatment (Figures 7, 8 and Table 2).

**TABLE 1:** Network topological characteristics in rhizosphere soils and bulk soils at the maize silking stage under different N fertilizer treatments.

Topological properties	Rhizosphere soil						Bulk soil					
		
	CT	U	S	SU3	SU5	SU7	CT	U	S	SU3	SU5	SU7
Nodes	45	35	44	48	41	45	48	45	48	44	48	48
Total links	282	204	279	341	268	338	375	420	351	355	379	377
Average clustering coefficient	0.975	0.964	0.97	0.973	0.97	0.975	0.979	0.98	0.975	0.976	0.976	0.978
Average degree	12.533	11.657	12.682	14.208	13.073	15.022	15.625	18.667	14.625	16.136	15.792	15.708
Average weighted degree	0.311	18.343	0.818	3.083	5.61	2.622	2.083	5.822	1.917	4.455	3.083	1.75
Network diagram density	0.285	0.343	0.295	0.302	0.327	0.341	0.332	0.424	0.311	0.375	0.336	0.334

*The different N fertilizer treatments include no fertilizer (CT), regular urea (U), controlled-release urea (S), and different ratios of controlled-release urea to regular urea (SU3, 3:7 ratio of controlled-release urea to regular urea; SU5, 5:5 ratio of controlled-release urea to regular urea; and SU7, 7:3 ratio of controlled-release urea to regular urea). Regular urea, controlled-release urea, and different ratios of regular urea and controlled-release urea are all applied at a rate of 180 kg ha^–1^.*

**TABLE 2:** Network topological characteristics in rhizosphere soils and bulk soils at the maize physiological maturity under different N fertilizer treatments.

Topological properties	Rhizosphere soil						Bulk soil					
		
	CT	U	S	SU3	SU5	SU7	CT	U	S	SU3	SU5	SU7
Nodes	47	54	49	49	45	46	43	47	46	55	47	56
Total links	383	383	428	353	326	394	307	314	353	712	453	504
Average clustering coefficient	0.976	0.973	0.976	0.976	0.972	0.979	0.975	0.973	0.979	0.986	0.98	0.982
Average degree	16.298	14.185	17.469	14.408	14.489	17.13	14.279	13.362	15.348	25.891	19.277	18
Average weighted degree	0.979	0.852	1.429	6.082	1.2	11.739	1.442	1.745	4.522	2.8	1.319	0.429
Network diagram density	0.354	0.268	0.364	0.3	0.329	0.381	0.34	0.29	0.341	0.479	0.419	0.327

*The different N fertilizer treatments include no fertilizer (CT), regular urea (U), controlled-release urea (S), and different ratios of controlled-release urea to regular urea (SU3, 3:7 ratio of controlled-release urea to regular urea; SU5, 5:5 ratio of controlled-release urea to regular urea; and SU7, 7:3 ratio of controlled-release urea to regular urea). Regular urea, controlled-release urea, and different ratios of regular urea and controlled-release urea are all applied at a rate of 180 kg ha^–1^.*

### Relationships Between the Microbial Network Complexity and the Maize Yield Traits and N Agronomic Efficiency and N Partial Factors Productivity

**FIGURE 9:**
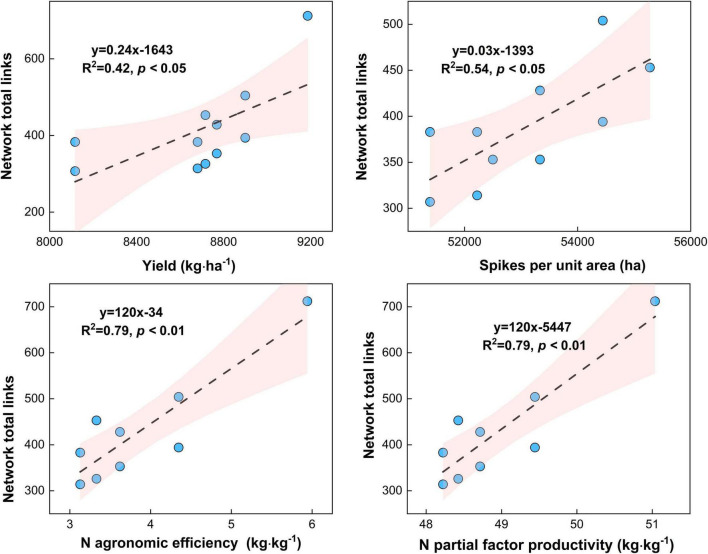
Relationship between microbial network complexity with maize yield, spikes per unit area, N agronomic efficiency, and N partial factor productivity under different N fertilizer treatments (including CT, U, S, SU3, SU5, and SU7).

Maize yield (*R*^2^ = 0.45, *p* < 0.05) and spikes per unit area (*R*^2^ = 0.54, *p* < 0.05) increased with the microbial network complexity (i.e., total links) under different urea treatments (Figure 9, *p* < 0.05). This result highlights that the high microbial network complexity (i.e., microbial network total links) is closely associated with the higher maize yield. Meanwhile, the N agronomic efficiency (*R*^2^ = 0.79, *p* < 0.05) and N partial factor productivity (*R*^2^ = 0.79, *p* < 0.01) increased with the microbial network complexity (i.e., total links) under different urea treatments (Figure 9, *p* < 0.05). This result highlights that the high microbial network complexity (i.e., microbial network total links) can enhance the N agronomic efficiency and N partial factor productivity.

## Discussion

### Effect of N Fertilizer Treatments on Maize Yield and Related Traits

Previous studies have shown that N fertilizer application can significantly contribute to increased maize yields (Zheng et al., 2016; Guo et al., 2017). In line with these studies, the results show that both regular and controlled-release urea alone and in combination can increase maize yield, spikes per area, and grain number per spike compared to the control treatment (Figure 1). However, the effects of N fertilizer on maize yield and spikes per area changed with the controlled-release urea and regular urea applied alone and their blending rates (Figure 1; Guo et al., 2021). Compared to the regular urea, the S, and SU3, both SU5 and SU7 can improve the maize yield (Figure 1). This might be due to the (i) excessive volatilization or loss of regular urea with precipitation leading to a potential loss in its contribution to maize growth and yield (Zhang et al., 2019); (ii) controlled-release urea is released into the soil solution at a rate that better matches the growth requirements of the crop and has a long fertilizer supply period (Shaviv, 2001; Li R. et al., 2021); and (iii) controlled-release urea could more efficiently improve the N use efficiency than regular urea (Zheng et al., 2016; Li et al., 2020). In this study, the SU3 treatment had the highest maize yield among the different blend ratios of regular and controlled-release urea (Figure 1). A similar result was confirmed by Guo et al. (2017, 2021), who found that the 3:7 blend ratio of controlled-release urea and regular urea was most effective in improving maize yield and NUE and mitigating NH_3_ volatilization and soil NO_3_-N leaching in Shaanxi province of China and Northwest China, respectively. This may be due to the fact that this ratio of controlled-release urea to regular urea both meets the nutrient requirements of maize in the early stages of fertility and provides a stable supply of nutrients for growth in the middle and late stages of fertility (Gao et al., 2021). Additionally, the N agronomic efficiency and N partial factor productivity were highest in the SU3 treatment, nearly 2 times and 1.2 times higher than the CT treatment, respectively (Figure 1, *p* < 0.05). This result suggests that SU3 treatment is more effective in improving N agronomic efficiency and N partial factor productivity than CT and other treatments. This is probably because this favorable ratio of controlled-release to regular urea both avoids rapid N losses and matches the fertilizer requirements of the maize growth stage, thus improving the N agronomic efficiency and N partial factor productivity (Zhang et al., 2022). Therefore, those results suggest that a 3:7 (SU3) blend of controlled-release urea and regular urea is the best ratio that maximizes summer maize yields and N agronomic efficiency in the North China Plain (Hebei Province).

### Effect of N Fertilizer Treatments on Microbial Diversity, Composition, and Network Complexity

Soil microorganisms mediate many important biological processes for sustainable agriculture and it is widely recognized that fertilization activities have an impact on the community structure of microorganisms (Hamer et al., 2009; Geisseler and Scow, 2014; Sun R. et al., 2021). For example, Hamer et al. (2009) found that urea fertilization induced the alteration in microbial community composition from Gram-positive to Gram-negative bacteria and fungi. The microbial composition involved in the N-transforming process was altered by fertilization (Sun R. et al., 2021). However, in a wide range of soil and environmental conditions in western Canada, fertilizer application (e.g., controlled-release urea) had no significant effects on soil microorganisms in most cases, and the few significant effects were increases in microbial biomass or functional diversity (Lupwayi et al., 2010). In line with Lupwayi et al. (2010), those results show that different ratios of controlled-release urea and regular urea application had less effect on changes in microbial community composition, which did not change significantly at the silking and maturity stages of maize (Figure 3). This result demonstrated that the short-term applications of regular urea and controlled-release urea in the North China Plain did not alter the composition of soil microbial communities, and these fertilizers did not adversely affect most soil biological processes (Lupwayi et al., 2010). In addition, over 10 years (long-term) experiments also suggested that the N inputs on soil microbial community structure were minor and slightly different between prokaryotes and fungi (Tosi et al., 2021). This may be due to the fact that some microbial taxa can withstand quite challenging conditions (e.g., fertilization), which may play an important role in the ecological adaptation of microorganisms and plant growth (De Vries and Shade, 2013). Thus, microbial communities that maintain a stable pattern in response to fertilization may help to maintain food and fiber production in the presence of long-term nutrient fertilization (Fan et al., 2020a).

Although the application of controlled-release urea and regular urea and their different ratios did not alter the microbial community composition of the maize rhizosphere and bulk soil, which significantly affected microbial α diversity (Figure 2), the result showed that the microbial diversity (i.e., Shannon, Simpson, Chao1, and ACE indexes) remained stable at the silking stage of maize with control and different normal and slow-release N fertilizer ratios, whereas that increased at the maize physiological maturity stage with the increasing slow-release to regular N fertilizer ratios (from SU3 to SU7; Figure 2). This result suggests that both regular and controlled-release urea alone and in combination can contribute to an increase in microbial diversity. This may be due to the reduced leaching of soil N caused by the combined application of controlled-release urea and regular urea, which still increased soil NO_3_^–^-N and NH_4_^+^-N content in late crop growth (Zheng et al., 2016). The reduction in NO_3_^–^-N and NH_4_^+^-N uptake by the crop in late growth facilitated the use of these soluble nutrients by microorganisms, which in turn stimulated an increase in microbial biomass and diversity (Lupwayi et al., 2010; Huang et al., 2021). However, a previous study showed that the bacterial and fungi α diversity in a blend of regular to controlled-release urea treatment were lower than the regular urea treatment (Li B. B. et al., 2021). This study contradicts our findings, suggesting that the effect of the combination of controlled-release and conventional urea on microbial diversity may be influenced by regional conditions, such as nutrient availability or changes in the crop fertility stage (Figure 2; Zhang et al., 2022). For example, when nutrient availability in the soil decreases or when reduced nutrient levels may limit microbial proliferation and nutrient mineralization, competition between crops and microbes for N may reduce microbial diversity (Inselsbacher et al., 2010). Lupwayi et al. (2010) found that the controlled-release urea increased MBC or functional diversity more than urea in 3 site-years, but the opposite was observed in 1 site-year. This result shows that the effect of controlled-release urea and normal urea on microbial communities is influenced by the age of application of the fertilizer.

In the maize silking stage, the microbial network total links and an average degree in treatments of the combination of regular and controlled-release urea were higher than the regular and slow-release N fertilizer applied separately (Figure 5 and Table 1). Simultaneously, the microbial network total links increased with the controlled-release to regular urea ratios (Figure 8 and Table 2). This result demonstrated that the combination of regular and controlled-release urea is more effective in improving microbial network complexity than the traditional regular and slow-release N fertilizer applied separately. This is because the microbial network complexity is influenced by the type of fertilizer and the amount of fertilizer applied (Ji et al., 2021; Li B. B. et al., 2021). The blending of controlled-release urea and regular urea affects its network complexity by influencing the diversity of microorganisms. The SU3 treatment had the highest microbial network complexity in this study among the different blend ratios of regular and controlled-release urea (Figure 8). This result indicates that the SU3 treatment is the optimal treatment to improve the complexity of the soil microbial network in the North China Plain.

### Relationships Between the Maize Yield and Microbial Network Complexity

Previous studies have shown that more complex systems with higher diversity are more productive because they are more resistant to environmental change (De Vries and Shade, 2013; Yuan et al., 2021). Additionally, the microbial network complexity enhances the link between biodiversity and multifunctionality in agricultural systems (Jiao et al., 2021). For example, the combination of inorganic fertilizer application and cow manure resulted in the most complex microbial community, and this network complexity was closely linked to high plant productivity and nutrient availability (Fan et al., 2020a). In this study, the application of controlled-release urea and regular urea and their different ratios resulted in more or fewer changes in microbial network complexity (Figure 5–8). Importantly, this result suggests that the maize yield increased with the microbial network complexity (i.e., total links) under different urea treatments (Figure 9, *p* < 0.05). The application SU3 led to the most resistant (i.e., higher total links) microbial community, which was associated with the highest levels of maize yield (Figures 1, 8). This result highlights that the high microbial network complexity (i.e., microbial network total links) resulted in a higher maize yield. This is mainly due to the (i) high microbial community network complexity (i.e., the most resistant microbial community) may lead to higher availability of nutrients for plants, which facilitate plants getting more nutrients and less competition from microbial species (Fan et al., 2020a), and (ii) the reduction in the relative abundance of potential plant fungal pathogens (Van Der Heijden et al., 2008). Moreover, the N agronomic efficiency and N partial factor productivity increased with the microbial network complexity (i.e., total links) under different urea treatments (Figure 9, *p* < 0.01). The SU3 treatment led to the most resistant (i.e., higher total links) microbial community, which was strongly associated with the highest levels of N agronomic efficiency and N partial factor productivity (Figures 1, 9). This result suggests that high microbial diversity and community complexity can significantly increase the N agronomic efficiency and N partial factor productivity of maize. This may be due to the fact that more complex microbial communities can accelerate soil nutrient cycling processes and enhance plant-microbial feedback (Fan et al., 2020a). Consequently, these findings suggest that the microbial network complexity is a critical factor in influencing maize production and fertilizer agronomic efficiency in an economically important crop system.

## Conclusion

This result suggests that the short-term applications of regular urea and controlled-release urea and their combinations in the North China Plain did not alter the composition of soil microbial communities, but they did contribute to an increase in microbial diversity. A combination of controlled-release and regular urea is more effective in improving maize yields than the regular and controlled-release urea applied separately (*p* < 0.05). In particular, in this study, the SU3 treatment had the highest maize yield (9,186 kg ha^–1^) among the different blend ratios of regular and controlled release urea, which increased by 13% in the SU3 treatment compared to the CT treatment. Simultaneously, the microbial network total links increased with the controlled-release to regular urea ratios, and the SU3 treatment had the highest microbial network complexity among the different blend ratios of regular and controlled-release urea. The SU3 treatment had the highest N agronomic efficiency and N partial factor productivity of all treatments, nearly 2 and 1.2 times higher than the CT treatment, respectively (*p* < 0.05). The N agronomic efficiency, N partial factor productivity, spikes per unit area, and maize yield increased with the microbial network complexity (i.e., total links). This result indicates that the SU3 treatment is the optimal treatment to both improve the maize yield and microbial network complexity in the North China Plain. Therefore, this study provides solid evidence that the microbial network complexity is essential for supporting maize yield under different N fertilizer treatments.

## Data Availability Statement

The data presented in the study are deposited in the Genome Sequence Archive (Genomics, Proteomics and Bioinformatics 2021) in National Genomics Data Center (Nucleic Acids Res 2022), China National Center for Bioinformation/Beijing Institute of Genomics, Chinese Academy of Sciences, accession numbers CRA005461 and CRA005462.

## Author Contributions

Y-CZ: conceptualization and funding acquisition, methodology, project administration, supervision, validation, and writing-review and editing. YP: sample collection. X-LL and P-JT: formal analysis and investigation. P-TJ: data curation, visualization, and writing-original draft. XD and J-cZ: data collection and correlation analysis of hormone data. All authors contributed to the article and approved the submitted version.

## Conflict of Interest

The authors declare that the research was conducted in the absence of any commercial or financial relationships that could be construed as a potential conflict of interest.

## Publisher’s Note

All claims expressed in this article are solely those of the authors and do not necessarily represent those of their affiliated organizations, or those of the publisher, the editors and the reviewers. Any product that may be evaluated in this article, or claim that may be made by its manufacturer, is not guaranteed or endorsed by the publisher.
